# Combination of ultrasound and molecular testing in malignancy risk estimate of Bethesda category IV thyroid nodules: results from a single-institution prospective study

**DOI:** 10.1007/s40618-021-01571-y

**Published:** 2021-04-16

**Authors:** M. Marina, M. C. Zatelli, M. Goldoni, P. Del Rio, L. Corcione, D. Martorana, A. Percesepe, F. Bonatti, P. Mozzoni, A. Crociara, G. Ceresini

**Affiliations:** 1 Dipartimento di Medicina e Chirurgia, SSD Medicina Interna Ad Indirizzo Onco-Endocrinologico, Università di Parma-Azienda Ospedaliero-Universitaria di Parma, Parma, Italy; 2 Dipartimento di Scienze Mediche, Sezione di Endocrinologia e Medicina Interna, UOL Endocrinologia-Università Degli Studi di Ferrara, Ferrara, Italy; 3 Dipartimento di Medicina e Chirurgia, Università di Parma, Parma, Italy; 4 UOC Clinica Chirurgica, Università di Parma-Azienda Ospedaliero-Universitaria di Parma, Parma, Italy; 5 UOC Anatomia e Istologia Patologica-Azienda Ospedaliero-Universitaria di Parma, Parma, Italy; 6 UOC Genetica, Università di Parma, Parma, Italy; 7 UOC Oncologia, Azienda Ospedaliero-Universitaria di Parma, Parma, Italy; 8 UOC Endocrinologia e Malattie del Ricambio, Azienda Ospedaliero, Universitaria di Ferrara, Ferrara, Italy

**Keywords:** Thyroid nodule, Ultrasound, Molecular, Markers, Fine-needle aspiration

## Abstract

**Purpose:**

Malignancy prediction in indeterminate thyroid nodules is still challenging. We prospectively evaluated whether the combination of ultrasound (US) risk stratification and molecular testing improves the assessment of malignancy risk in Bethesda Category IV thyroid nodules.

**Methods:**

Ninety-one consecutively diagnosed Bethesda Category IV thyroid nodules were prospectively evaluated before surgery by both ACR- and EU-TIRADS US risk-stratification systems and by a further US-guided fine-needle aspiration cytology (FNAC) for the following molecular testing: BRAFV600E, N-RAS codons 12/13, N-RAS codon 61, H-RAS codons 12/13, H-RAS codon 61, K-RAS codons 12/13, and K-RAS codon 61 point-mutations, as well as PAX8/PPARγ, RET/PC1, and RET/PTC 3 rearrangements.

**Results:**

At histology, 37% of nodules were malignant. No significant association was found between malignancy and either EU- or ACR-TIRADS. In total, 58 somatic mutations were identified, including 3 BRAFV600E (5%), 5 N-RAS 12/13 (9%), 13 N-RAS 61 (22%), 7 H-RAS 12/13 (12%), 11 H-RAS 61 (19%), 6 K-RAS 12/13 (10%), 8 K-RAS 61 (14%) mutations and 2 RET/PTC1 (4%), 0 RET/PTC 3 (0%), 3 PAX8/PPARγ (5%) rearrangements. At least one somatic mutation was found in 28% and 44% of benign and malignant nodules, respectively, although malignancy was not statistically associated with the outcome of the mutational test. However, the combination of ACR-, but not EU-, TIRADS with the presence of at least one somatic mutation, was significantly associated with malignant histology (*P* = 0.03).

**Conclusion:**

US risk stratification and FNAC molecular testing may synergistically contribute to improve malignancy risk estimate of Bethesda category IV thyroid nodules.

**Supplementary Information:**

The online version contains supplementary material available at 10.1007/s40618-021-01571-y.

## Introduction

Thyroid cancer is the most frequent endocrine tumor with an increasing incidence [1, 2], typically presenting as a thyroid nodule. However, most thyroid nodules are benign, with 5–15% malignancy risk [3–5], highlighting the crucial importance to estimate their malignancy risk to prevent patients to receive unnecessary surgery. Currently, the most reliable and common diagnostic procedure for thyroid nodule diagnosis is fine-needle aspiration cytology (FNAC) which accurately diagnoses benign and malignant nodules in most cases. However, in 10–25% of cases, nodules are cytologically diagnosed as indeterminate [6, 7] and frequently patients are submitted to surgery to obtain a histological diagnosis. Nevertheless, only a small proportion of indeterminate nodules is found to be malignant at histology with surgery being unnecessary in a considerable number of these patients [7–9].

The most challenging indeterminate nodules are those which, according to The Bethesda System for Reporting Thyroid Cytopathology (TBSRTC), are classified as atypia of undetermined significance/follicular lesion of undetermined significance (AUS/FLUS) (Bethesda Category III), and follicular or oncocytic (Hürthle cell) neoplasm/suspicious for a follicular or oncocytic (Hürthle cell) neoplasm (FN/SFN) (Bethesda Category IV) [10–12]. The 2017 TBSRTC edition [13] revised the predicted probability of malignancy for indeterminate nodules which was estimated to be 10–30 and 25–40% for AUS/FLUS and FN/SFN, respectively, when the noninvasive follicular thyroid neoplasm with papillary-like nuclear features (NIFTP) is considered as a malignant tumor. While for AUS/FLUS nodules, the possibility to follow-up patients and repeat FNAC has been recommended [14], FN/SFN nodules represent a more relevant clinical problem to solve.

Molecular testing on FNAC material has been employed in the diagnosis of thyroid nodules to better define the risk of malignancy and, in 2009, Nikiforov and co-workers demonstrated the feasibility of a molecular testing panel, including BRAF and RAS mutations as well as RET/PTC and PAX8/PPARγ rearrangements [15]. Other subsequent reports have strengthened the importance of this approach, which was demonstrated to improve the assessment of malignancy risk of indeterminate nodules by means of mutational and gene expression analyses [16, 17]. However, the uncertainty in defining malignancy risk of indeterminate thyroid nodules is not completely solved at present.

In the past few years, ultrasound (US)-based risk estimates for thyroid nodules have been developed. The Thyroid Imaging Reporting and Data System (TIRADS) has been established and endorsed by the American College of Radiologists (ACR-TIRADS) [18], the European Thyroid Association (EU-TIRADS) [19], and the Korean Society of Thyroid Radiology (K-TIRADS) [20] to reduce unnecessary thyroid nodule FNAC. Therefore, the TIRADS is increasingly becoming the standard type of US reporting of thyroid nodules. However, US malignancy risk estimate of indeterminate nodules is still a matter of discussion. Indeed, AUS/FLUS and FN/SFN classes may include a significant proportions of follicular thyroid cancers (FTC) and follicular variant of papillary thyroid cancer (FVPTC); these histological subtypes can have unsuspicious US presentation [21, 22].

Little is known on whether US characteristics of indeterminate thyroid nodules may concur to better define the risk estimate when assessed in association with molecular testing. In this study, we prospectively evaluated whether the combination of US risk-stratification systems and molecular testing improves the assessment of malignancy risk in TBSRTC Category IV thyroid nodules.

## Materials and methods

We prospectively evaluated 106 thyroid nodules in euthyroid patients consecutively referred to the Unit of Internal Medicine and Oncological Endocrinology of the University of Parma-University Hospital of Parma from Nov 2014 to 2018 with a cytological diagnosis of category IV thyroid nodule, according to the 2010 TBSRTC. All patients had already been referred to surgeon for lobectomy or total thyroidectomy. In almost all cases, FNAC was performed at the same institution with cytological reading performed at the local pathology unit. All cytological readings were reviewed by an expert cytopathologist (LC). The study consisted in a US re-evaluation of all nodules and a subsequent FNAC for molecular testing.

### Ultrasound study

US was performed by an US machine (MyLab 70 X-Vision^®^-Esaote, Milan, Italy) with a 4–13 MHz probe. Nodule characteristics recorded during US examination were: diameters (anteroposterior, transverse, and longitudinal); shape, which was considered taller-than-wide if the anteroposterior diameter exceeded the transverse diameter; margins (smooth or ill-defined, including lobulated or irregular); composition (solid, cystic, and mixed); echogenicity (hyperechoic, isoechoic, hypoechoic—relative to the thyroid parenchyma—or markedly hypoechoic, i.e., more hypoechoic than strap muscles); calcifications (absent, microscopic, macroscopic—including rim calcifications); comet-tail artifacts and other indeterminate hyperechoic foci; vascularization (intranodular, peripheral or mixed) and suspected extrathyroidal extension. Each nodule was classified according to the widely used US risk-stratification systems ACR-TIRADS and EU-TIRADS. In addition, the ACR-TIRADS score was also calculated, according to the characteristics of this risk-stratification system, to be used as a continuous variable in the statistical analyses. US descriptions were assessed in real-time during the US evaluation; TIRADS were defined during the review of the recorded images. All US evaluations were performed by the same endocrinologist experienced in thyroid US (GC) and all images were recorded for further examination which was jointly performed by two endocrinologists experienced in thyroid US (GC and MM) to maximally reduce the impact of interobserver variability.

### FNAC for molecular testing

Nodules were then submitted to FNAC for molecular testing. FNAC was performed by capillarity using a 90 mm, 27-gauge needle (Artsana, Granate, Italy) equipped with a mandrel which was removed once the nodule was reached to selectively sample the nodule. The maneuver was performed under US guidance with a 10–5.5 probe (Esaote, Italy). Upon collection, each specimen was harvested in a 1.5 mL Eppendorf tube and immediately frozen in dry ice along with another Eppendorf tube containing 0.5 mL saline in which the needle was rinsed. Soon after, all tubes were frozen at − 80 °C for further molecular testing. All FNACs were performed by the same operator (MM). Two FNACs were performed for each nodule with the same procedure. The material from the second FNAC was used if the first one failed to obtain enough material for molecular testing.

### Molecular testing

#### BRAF and RAS mutation analyses

DNA isolation for BRAF and RAS somatic mutation analyses were performed at the Section of Endocrinology of the University of Ferrara, Italy, using the needle wash out. The presence of somatic BRAF and RAS mutations was investigated in each sample as previously described [23] using the EasyPGX^®^ ready THYROID CE IVD kit (cod. RT028, Diatech Pharmacogenetics srl, Jesi, Ancona, Italy) on a real-time PCR machine (CFX96 Touch Real-Time PCR Detection System, BioRad, Milano Italy), following the manufacturer’s instructions.

#### RNA extraction and cDNA synthesis for rearrangements

All the analyses for rearrangement identification were performed at the Institute of Genetics, and Laboratory of the Department of Medicine and Surgery, University Hospital of Parma, Italy. Total RNA was extracted using the RNeasy^®^ Plus Mini Kit (Qiagen) following the manufacturer’s instructions. Concentration and purity of each RNA sample were assessed using Nanodrop spectrometer (Thermo Scientific, Wilmington, DE) and RNA integrity score was obtained by Tapestation (Agilent Technologies, Wilmington, DE). Purified RNA samples were stored at − 80 °C until analysis. Reverse transcription was carried out using the QuantiTect^®^ Reverse Transcription Kit (Qiagen) according to the manufacturer’s instructions. Samples were stored at − 80 °C. For Real-time PCR cDNA was amplified using the iCycler iQ Real-Time Detection System (BioRad, CA, USA) with the TaqMan 2 × Universal PCR Master Mix with No AmpErase UNG (Life Technologies, CA, USA). For the identification of the PAX8/PPARγ gene rearrangement, a pre-designed TaqMan probe was used (Assay ID: Hs04396712_ft, ThermoFisher Scientific, USA). For RET/PTC1 and RET/PTC3 gene rearrangements, custom Dual Labeled Probes were used (Metabion International AG) (Supplementary Table 1). Finally, for the TaqMan Human Endogenous Control, PGK1 housekeeping gene (Thermo Fisher Scientific, USA) was used. Quantification was performed by employing the ^2−^∆∆Ct method [24].

The study was double blinded: molecular testing personnel was blinded to all FNAC, US, and histopathology data, and pathologists were blinded to molecular testing, FNAC, and US findings. The study was performed in accordance with the ethical standards according to the 1964 Declaration of Helsinki and its later amendments. A written informed consent was obtained from each patient before enrollment. The study was approved by the Ethics Committee of Parma (protocol N.25116; 07-14-2014).

### Statistical analysis

Qualitative variables are expressed as absolute and percentage frequency and differences between them were assessed by means of Chi squared or Fisher exact test depending on the frequency. All the variables were tested with univariate models of logistic regression to find crude significances and Odds Ratio. The normality of quantitative variables was assessed by means of Kolmogorov–Smirnov test. In the case of normality, data were reported as mean (M) ± standard deviation (SD). Otherwise, median and interquartile ranges (IQ) were reported. Consequently, independent sample *t* test or Mann–Whitney *U* test were used. Correlations between variables were assessed using Pearson’s correlation test. Multivariate models of logistic regression were used to test possible independent factors influencing histological results, including in the model all the possible variables with clinical meaning independently of their univariate significance and excluding multicollinearity. Finally, sensitivity, specificity, positive predictive value (PPV), and negative predictive value (NPV) were calculated. A *P* value ≤ 0.05 was chosen as significant. For all the analyses, IBM SPSS Statistics v 26 (IBM, Amork, NY, USA) was used.

## Results

Of the 106 nodules enrolled, 10 were excluded because the patients chose to postpone surgery, 4 because the final histology was not consistent with thyroid lesions (i.e., 3 parathyroid adenoma, 1 paraganglioma), 1 because of poor quality of nucleic acids. Therefore, data presented here are from 91 FN/SFN nodules collected in 90 patients, whose demographic characteristics are reported in Table 1. Patients were more likely to be female and the mean maximum nodule diameter was 22 mm. US characteristics are described in Table 1. When considering the ACR-TIRADS classification system, the most represented class was “TR4 - Moderately suspicious” which was observed in 47% of cases. “High risk” was the most frequent class when considering EU-TIRADS classification, being observed in 42% of cases (Table 2).

**Table 1:** General characteristics of the nodules

Age of the patients M (SD)	54 (12.9)
Sex of the patients F (%)	65 (71)
Maximum US nodule diameter mm, M (SD)	22 (12)
Maximum histologic diameter mm, M (SD)	21 (12)
ACR score M (SD)	4.64 (2.1)
Hypoechoic nodule *n* (%)	55 (60)
Isoechoic nodule *n* (%)	36 (40)
Hyperechoic nodule *n* (%)	0 (0)
Solid nodule *n* (%)	78 (86)
Mixed nodule *n* (%)	13 (14)
Irregular margins *n* (%)	16 (18)
Microcalcifications *n* (%)	13 (14)
Taller than wide *n* (%)	10 (11)
US findings of extrathyroidal extension	2 (2)
Histological type	
FVPTC	26 (28)
FTC	6 (7)
MTC	2 (2)
Benign histology *n* (%)	57 (63)
Histological type	
Follicular adenoma	45 (50)
Adenomatous hyperplasia	12 (13)
Multinodular goiter	73 (80)

*M* mean, *(SD)* standard deviation, *F* female, *US* ultrasound, *FVPTC* follicular variant of papillary thyroid cancer, *FTC* follicular thyroid cancer, *MTC* medullary thyroid cancer

**Table 2:** General US (A) and molecular characteristics (B) of the nodules

A			
ACR-TIRADS *n* (%)	TR1—benign	0 (0)	
	TR2—not suspicious	10 (11)	
	TR3—mildly suspicious	13 (14)	
	TR4—moderately suspicious	43 (47)	
	TR5—highly suspicious	25 (28)	

EU-TIRADS *n* (%)	Benign	0 (0)	
	Low risk	28 (31)	
	intermediate risk	25 (27)	
	High risk	38 (42)	

B			
Total number of nodules with molecular alterations *n* (%)	31 (34)		

Molecular alterations	*N*	% of total molecular alterations	% of total nodules
BRAF V600E	3	5	3
N-RAS 12–13	5	9	5
N-RAS 61	13	22	14
H-RAS 12–13	7	12	8
H-RAS 61	11	19	12
K-RAS 12–13	6	10	7
K-RAS 61	8	14	9
RET/PTC 1	2	4	2
RET/PTC 3	0	0	0
PAX8/PPARγ	3	5	3
Total	58		

*US* ultrasound

We found at least one molecular alteration in 31 nodules (34%), for a total of 58 molecular derangements. The lowest prevalence was found for BRAFV600E, PAX8/PPARγ, and RET/PTC1, while RET/PTC3 rearrangement was not present. The most present were H-RAS 61 and N-RAS 61 somatic mutations (Table 2).

Malignant histology was found in 37% of cases with FVPTC being the most represented histological type (Table 1). Among the investigated nodules, two were consistent with NIFTP and were considered among the group of FVPTC. FTC was found in six cases (7%), five of which were minimally invasive, and one was widely invasive. Follicular adenoma was the most frequently observed benign histology. Differences in US characteristics and molecular testing between benign and malignant nodules are shown in Table 3.

**Table 3:** US characteristics and molecular markers in benign and malignant nodules

	Benign *n* 57	Malignant *n* 34	P*
ACR-TIRADS *n* (%)			
TR1—benign	0 (0)	0 (0)	0.33
TR2—not suspicious	6 (10)	4 (12)	
TR3—mildly suspicious	9 (16)	4 (12)	
TR4—moderately suspicious	30 (52)	13 (38)	
TR5—highly suspicious	12 (21)	13 (38)	
ACR-TIRADS score	4.5 (1.8)	4.9 (2.6)	0.39
EU-TIRADS *n* (%)			
Benign	0 (0)	0 (0)	0.99
Low risk	17 (30)	11 (32)	
Intermediate risk	16 (28)	9 (27)	
High risk	24 (42)	14 (41)	
Nodules with at least one molecular alteration	16 (28)	15 (44)	0.17
Molecular alterations (% on number of nodules)			
BRAF V600E	0 (0)	3 (9)	
N-RAS 12–13	5 (9)	0 (0)	
N-RAS 61	8 (14)	5 (15)	
H-RAS 12–13	5 (9)	2 (6)	
H-RAS 61	8 (14)	3 (9)	
K-RAS 12–13	5 (9)	1 (3)	
K-RAS 61	7 (12)	1 (3)	
RET/PTC1	2 (4)	0 (0)	
RET/PTC3	0 (0)	0 (0)	
PAX8/PPARγ	2 (4)	1 (3)	

*US* ultrasound

^*^Fisher exact test

Regarding the ACR-TIRADS, benign nodules were mostly represented by the “TR4 moderately suspicious” class (52%), whereas “TR3 mildly suspicious” and “TR5-highly suspicious” equally represented the most frequent class (38%) in malignant nodules. Regarding the EU-TIRADS, the most representative class was the “High risk” one, with 42% and 41% frequency in benign and malignant nodules, respectively. Overall, US characteristics did not significantly differ between benign and malignant nodules. Therefore, these systems did not accurately separate benign from malignant nodules in our series.

The frequency of molecular alterations was higher in malignant nodules (44%) as compared to benign nodules (28%), but this difference did not reach statistical significance. Among molecular markers, N-RAS 61, H-RAS 61, and K-RAS 61 somatic mutations mainly characterized benign nodules, whereas N-RAS 61 was the main molecular alteration identified among malignant nodules (15%). However, its frequency was similar to that found in benign nodules (14%). All BRAFV600E mutated nodules were malignant. Among 60 mutation-negative samples, 41 (68%) were benign and 19 (31%) were malignant.

Considering nodules with at least one molecular alteration, we did not find any significant association with US characteristics, described by either ACR-TIRADS or EU-TIRADS risk assessment (Chi-square value: 1.101 and 0.224, respectively; *P* value: 0.294 and 0.636, respectively). Only one tumor sample harbored multiple mutations; in particular, this sample displayed the H-RAS 12–13 and the BRAFV600E somatic mutations and was consistent with a FVPTC at histology.

The relationships between US characteristics, mutational test outcome and malignant histology are reported in Table 4a. Malignancy was not associated with the outcome of the mutational test, nor with US risk assessment by ACR- and EU-TIRADS. However, ACR-TIRADS displayed the best performance. Therefore, we run a univariate analysis, testing the combination of ACR-TIRADS risk stratification plus the presence of at least one molecular alteration versus malignant histology. The results, reported in Table 4b, demonstrated that this combination was significantly associated to malignant histology (*P* = 0.03), with a sensitivity of 65% (95% CI 46–80), specificity of 58% (95% CI 44–71), PPV of 48% (95% CI 33–63), and NPV of 73% (95% CI 58–85). EU-TIRADS was far from reaching statistical significance at the univariate analysis (Table 4a), therefore, it was not considered for the US + molecular testing combination analysis.

**Table 4:** Relationship between US characteristics, molecular markers and malignant histology (univariate analysis)

A	Beta	SE	*P*	OR	OR 95% CI
Molecular marker alterations	0.705	0.454	0.121	2.023	0.831–4.926
EU-TIRADS	− 0.088	0.435	0.839	0.915	0.390–2.148
ACR-TIRADS	0.806	0.472	0.088	2.238	0.887–5.644
ACR-TIRADS score	0.089	0.103	0.385	1.093	0.894–1.337

B	Beta	SE	*P*	OR	OR 95% CI
ACR-TIRADS plus at least one molecular marker alteration	0.925	0.448	0.039	2.521	1.048–6.066

*SE* standard error, *OR* odds ratio, *US* ultrasound

## Discussion

In this study, we found that the combination of US risk-stratification systems and molecular testing improves the assessment of malignancy risk in TBSRTC IV thyroid nodules as compared to each individual assessment.

In line with the data reported by TBSRTC [13], we found a malignancy rate of 37% in our series of FN/SFN nodules, indicating that the latter was correctly characterized, avoiding selection bias. NIFTP may represent a significant proportion of AUS/FLUS and FN/SFN classes. However, in our series, we found only two NIFTP (2.2% of the whole group), representing a very low proportion of the identified lesions. On this background, we decided to keep these two cases in the “malignant” group of FVPTC, not to lose statistical power in the different groups.

The role of US in the risk estimate of cytologically indeterminate thyroid nodules is a matter of debate. We found a quite high percentage of high-risk TIRADS categories among benign tumors, reducing the potential of US characteristics to detect malignant nodules, despite the correct use of these US risk estimation systems, as indicated by the literature [18, 19]. Hypoechogenicity represents the US feature that accounts for these specificity issues. Hypoechogenicity was frequently found in benign as well as malignant nodules in our series. This may represent a confounding factor that impairs the identification of malignancy. Regarding the performance of US risk-stratification in cytologically indeterminate thyroid nodules, Trimboli et al. reported a suboptimal accuracy, although high sensitivity was found for the American Thyroid Association (ATA) system [23, 24]. This issue is of particular interest when dealing with FTC whose cytology at FNAC examination is included within TBSRTC indeterminate categories. US does not provide specific markers for FTC and, although different US criteria have been suggested [25, 26], its role remains questioned [27]. More promising appear to be the findings of Grani and co-workers who reported encouraging results from ATA and TIRADS on the risk estimate of indeterminate nodules [28]. However, most of these data are based on retrospective analyses. Moreover, the indeterminate classes are often reported according to different classification systems. The novelty of our study is represented by the fact that US was performed after a diagnostic FNAC in a prospective design and by selectively targeting FN/SFN nodules.

As compared to the EU-TIRADS, which we included in our US evaluation based on the geographic area of our patients, we found that ACR-TIRADS provides a better performance, even though it has no predictive value for malignancy.

To improve the risk stratification of indeterminate thyroid nodules several studies tested the diagnostic potential of somatic mutation panels in thyroid FNAC material [15, 16]. Large genetic panels, such as ThyroSeq v2 next-generation sequencing (NGS) assay have been developed. Nikiforov and co-workers studied 104 benign and 39 malignant FN/SFN nodules, with a sensitivity of 90%, specificity of 93%, PPV of 83% and NPV of 96% [12]. Valderrabano and co-workers reported a slightly worse performance in a series of 37 benign and 13 malignant FN/SFN nodules, with a sensitivity of 85%, specificity of 84%, PPV of 65%, and NPV of 94% [29]. In 2019, Steward and co-workers reported the results of the ThyroSeq v3 Genome Classifier on the malignancy risk estimate of 60 benign and 33 malignant at TBSRTC IV nodules, with a sensitivity of 97%, specificity of 75%, NPV of 98%, and PPV of 68% [30]. However, commercial panels for molecular testing are not always available outside research protocols, are generally expensive [31], and therefore, may not be easily affordable in clinical practice.

In our study, we used a noncommercial panel which largely derives from previous experiences [32, 33]. We found a molecular alteration in 34% of nodules, half of which had malignant histology. These data differ from previous results reported with noncommercial panels. Using a 7-gene panel, including BRAF, RAS, RET/PTC, and PAX8/PPARγ, on a total of 214 FN/SFN nodules, Nikiforov and co-workers demonstrated mutational positivity in 18% of samples, 87% of which were malignant at histology [34]. With a wider mutational panel, Beaudenon-Huibregtse and co-workers demonstrated a mutational positivity in 26% of 19 TBSRTC IV nodules, finding a molecular alteration in 4 out of 6 malignant nodules and in 1 out of 13 benign nodules [35]. In 2017, Eszlinger and co-workers studied 199 FN/SFN nodules from a European population using a panel including BRAF and RAS mutations as well as PAX8/PPARγ and RET/PTC rearrangements. They found a mutational positivity in 11% of cases with the detection of 8/30 carcinomas, while 14/156 benign samples revealed a false-positive test. Their sensitivity was 27% with a specificity of 91%, a PPV and a NPV of 36 and 87%, respectively [36]. Therefore, our data may be included in the spectrum of the wide performance variability of studies employing noncommercial tests, which is possibly due to different study design (including the fact that some reports are from retrospective studies), nodule number and size, and genetic background of the studied populations.

In our series, we found a higher mutation rate as compared to many of the previously published studies that used noncommercial panels. However, malignancy rate in our mutation positive cases is lower than that reported in other studies [34, 37], although higher than that documented by others [36]. Since the overall prevalence of malignancy in our series is comparable with that reported in the literature for TBSRTC IV nodules [13], a selection bias is unlikely to have occurred in our study. We can hypothesize that, based on our results, the genetic characteristics of FN/SFN nodules diagnosed in our population are different from those reported in other studies. More specifically, the presence of N-RAS 61, H-RAS 12–13, and PAX8/PPARγ genetic alterations in histologically benign nodules, in keeping with previous reports [38, 39] could have affected the specificity of our results. This does not apply to BRAF mutations; all cases corresponded to a malignant histology, in line with the high prediction of malignancy of this molecular marker [32, 37]. We cannot exclude that the limited sample size may have affected the sensitivity of our study, which is relatively low, although higher than that reported by the other authors using similar molecular testing [36]. Of note, we also analyzed our results after selecting only the 7 genes used in previous studies [34, 36] out of the 10 genes included in our panel: we did not find any association between malignant histology and molecular test outcome alone or molecular test outcome plus US findings (data not shown).

Points of strength of our study are represented by the monocentric, prospective, and double-blinded design using the international Bethesda classification system for categorizing our cytology, according to the ATA guidelines. Moreover, US studies and FNAC have all been performed by the same persons. Cytology and histology have been carried out at the same Institution by the same team of pathologists. Point of weakness may be represented by the limited nodule sample size. However, the main feature of this study is of methodological interest. In fact, we tried to verify whether, in a prospective study, the combination between the new US reporting systems with molecular testing may improve the risk estimate of FN/SFN thyroid nodules. To the best of our knowledge, this is the first study based on such approach and with such design.

Although our approach is characterized by quite low sensitivity and specificity, our strategy allows for an improvement in these parameters. Validation studies with higher number of nodules are needed to define the role of this combination strategy in the decision making to operate or not an indeterminate thyroid nodule. In this context, our approach may be considered as a pilot study for further experience aimed at extending the number of observations on this issue, to impact on practical approach.

## Supplementary Information

Below is the link to the electronic supplementary material.

Supplementary file1 (DOCX 14 KB)

## Data Availability

Upon request to: Graziano Ceresini, MD, PhD, corresponding author.

## Code availability

Upon request to: Graziano Ceresini, MD, PhD, corresponding author.

## References

[1] Ceresini G, Corcione L, Michiara M, Sgargi P, Teresi G, Gilli A, Usberti E, Silini E, Ceda GP (2012). Thyroid cancer incidence by histological types and related variants in a mildly iodine-deficient area of Northern Italy, 1998–2009. Cancer.

[2] Lim H, Devesa SS, Sosa JA, Check D, Kitahara CM (2017). Trends in thyroid cancer incidence and mortality in the United States, 1974–2013. JAMA.

[3] Cooper DS, Doherty GM, Haugen BR, Kloos RT, Lee SL, Mandel SJ, Mazzaferri EL, McIver B, Sherman SI, Tuttle RM (2006). Management guidelines for patients with thyroid nodules and differentiated thyroid cancer. Thyroid.

[4] Frates MC, Benson CB, Doubilet PM, Kunreuther E, Contreras M, Cibas ES, Orcutt J, Moore FD, Larsen PR, Marqusee E, Alexander EK (2006). Prevalence and distribution of carcinoma in patients with solitary and multiple thyroid nodules on sonography. J Clin Endocrinol Metab.

[5] Kim DL, Song KH, Kim SK (2008). High prevalence of carcinoma in ultrasonography-guided fine needle aspiration cytology of thyroid nodules. Endocr J.

[6] Gharib H (2004). Changing trends in thyroid practice: understanding nodular thyroid disease. Endocr Pract.

[7] Yassa L, Cibas ES, Benson CB, Frates MC, Doubilet PM, Gawande AA, Moore FD, Kim BW, NoseMarquseeLarsenAlexander VEPREK (2007). Long-term assessment of a multidisciplinary approach to thyroid nodule diagnostic evaluation. Cancer.

[8] Baloch ZW, Fleisher S, LiVolsi VA, Gupta PK (2002). Diagnosis of “follicular neoplasm”: a gray zone in thyroid fine-needle aspiration cytology. Diagn Cytopathol.

[9] Mazzaferri EL (1993). Management of a solitary thyroid nodule. N Engl J Med.

[10] Ali SZ, Cibas ES (2010). The Bethesda system for reporting thyroid cytopathology.

[11] Baloch ZW, LiVolsi VA, Asa SL, Rosai J, Merino MJ, Randolph G, Vielh P, DeMay RM, Sidawy MK, Frable WJ (2008). Diagnostic terminology and morphologic criteria for cytologic diagnosis of thyroid lesions: a synopsis of the National Cancer Institute Thyroid Fine-Needle Aspiration State of the Science Conference. Diagn Cytopathol.

[12] Nikiforov YE, Carty SE, Chiosea SI, Coyne C, Duvvuri U, Ferris LR, Gooding WE, Hodak SP, LeBeau SO, Ohori NP, Seethala RR, Tublin ME, Yip L, Nikiforova MN (2014). Highly accurate diagnosis of cancer in thyroid nodules with follicular neoplasm/suspicious for a follicular neoplasm cytology by ThyroSeq v2 next-generation sequencing assay. Cancer.

[13] Cibas ES, Ali SZ (2017). The 2017 Bethesda system for reporting thyroid cytopathology. Thyroid.

[14] Haugen BR, Alexander EK, Bible KC, Doherty GM, Mandel SJ, Nikiforov YE, Pacini F, Randolph GW, Sawka AM, Schlumberger M, Schuff KG, Sherman SI, Sosa JA, Steward DL, Tuttle RM, Wartofsky L (2015). American Thyroid Association Management Guidelines for adult patients with thyroid nodules and differentiated thyroid cancer: The American Thyroid Association Guidelines Task Force on Thyroid Nodules and Differentiated Thyroid Cancer. Thyroid.

[15] Nikiforov YE, Steward DL, Robinson-Smith TM, Haugen BR, Klopper JP, Zhu Z, Fagin JA, Falciglia M, Weber K, Nikiforova MN (2009). Molecular testing for mutations in improving the fine-needle aspiration diagnosis of thyroid nodules. J Clin Endocrinol Metab.

[16] Duick DS, Klopper JP, Diggans JC, Friedman L, Kennedy GC, Lanman RB, McIver B (2012). The impact of benign gene expression classifier test results on the endocrinologist-patient decision to operate on patients with thyroid nodules with indeterminate fine-needle aspiration cytopathology. Thyroid.

[17] Fagin JA, Wells SA (2016). Biologic and clinical perspectives on thyroid cancer. N Engl J Med.

[18] Tessler FN, Middleton WD, Grant EG, Hoang JK, Berland LL, Teefey SA, Cronan JJ, Beland MD, Desser TS, Frates MC, Hammers LW, Hamper UM, Langer JE, Reading CC, Scoutt LM, Stavros AT (2017). ACR thyroid imaging, reporting and data system (TI-RADS): white paper of the ACR TI-RADS Committee. J Am Coll Radiol.

[19] Russ G, Bonnema SJ, Erdogan MF, Durante C, Ngu R, Leenhardt L (2017). European Thyroid Association Guidelines for ultrasound malignancy risk stratification of thyroid nodules in adults: The EU-TIRADS. Eur Thyroid J.

[20] Shin JH, Baek JH, Chung J, Ha EJ, Kim JH, Lee YH, Lim HK, Moon WJ, Na DG, Park JS, Choi YJ, Hahn SY, Jeon SJ, Jung SL, Kim DW, Kim EK, Kwak JY, Lee CY, Lee HJ, Lee JH, Lee KH, Park SW, Sung JY (2016). Ultrasonography diagnosis and imaging-based management of thyroid nodules: revised Korean society of thyroid radiology consensus statement and recommendations. Korean J Radiol.

[21] Trimboli P, Deandrea M, Mormile A, Ceriani L, Garino F, Limone PP, Giovanella L (2018). American Thyroid Association ultrasound system for the initial assessment of thyroid nodules: use in stratifying the risk of malignancy of indeterminate lesions. Head Neck.

[22] Trimboli P, Fulciniti F, Zilioli V, Ceriani L, Giovanella L (2017). Accuracy of international ultrasound risk stratification systems in thyroid lesions cytologically classified as indeterminate. Diagn Cytopathol.

[23] Zatelli MC, Trasforini G, Leoni S, Frigato G, Buratto M, Tagliati F, Rossi R, Cavazzini L, Roti E, degli Uberti EC (2009). BRAF V600E mutation analysis increases diagnostic accuracy for papillary thyroid carcinoma in fine-needle aspiration biopsies. Eur J Endocrinol.

[24] Pfaffl MW, Bustin SA (2004). Quantification strategies in real-time PCR. The real-time PCR encyclopedia A-Z of quantitative PCR.

[25] Grani G, Lamartina L, Durante C, Filetti S, Cooper DS (2018). Follicular thyroid cancer and Hürthle cell carcinoma: challenges in diagnosis, treatment, and clinical management. Lancet Diabetes Endocrinol.

[26] Kobayashi K, Hirokawa M, Yabuta T, Masuoka H, Fukushima M, Kihara M, Higashiyama T, Ito Y, Miya A, Amino N, Miyauchi A (2016). Tumor protrusion with intensive blood signals on ultrasonography is a strongly suggestive finding of follicular thyroid carcinoma. Med Ultrason.

[27] Castellana M, Piccardo A, Virili C, Scappaticcio L, Grani G, Durante C, Giovanella L, Trimboli P (2020). Can ultrasound systems for risk stratification of thyroid nodules identify follicular carcinoma?. Cancer Cytopathol.

[28] Grani G, Lamartina L, Ascoli V, Bosco D, Nardi F, D'Ambrosio F, Rubini A, Giacomelli L, Biffoni M, Filetti S, Durante C, Cantisani V (2017). Ultrasonography scoring systems can rule out malignancy in cytologically indeterminate thyroid nodules. Endocrine.

[29] Valderrabano P, Khazai L, Leon ME, Thompson ZJ, MaZ CCH, Hallanger-Johnson JE, Otto KJ, Rogers KD, Centeno BA, McIver B (2017). Evaluation of ThyroSeq v2 performance in thyroid nodules with indeterminate cytology. Endocr Relat Cancer.

[30] Steward DL, Carty SE, Sippel RS, Yang SP, Sosa JA, Sipos JA, Figge JJ, Mandel S, Haugen BR, Burman KD, Baloch ZW, Lloyd RZ, Seethala RR, Gooding WE, Chiosea SI, Gomes-Lima C, Ferris RL, Folek JM, Khawaja RA, Kundra P, Loh KS, Marshall CB, Mayson S, McCoy KL, Nga MN, Ngiam KY, Nikiforova MN, Poehls JL, Ringel MD, Yang H, Yip L, Nikiforov YE (2019). Performance of a multigene genomic classifier in thyroid nodules with indeterminate cytology: a prospective blinded multicenter study. JAMA Oncol.

[31] Nishino M (2016). Molecular cytopathology for thyroid nodules: a review of methodology and test performance. Cancer Cytopathol.

[32] Nikiforov YE (2017). Role of molecular markers in thyroid nodule management: then and now. Endocr Pract.

[33] Muzza M, Colombo C, Pogliaghi G, Karapanou O, Fugazzola L (2020). Molecular markers for the classification of cytologically indeterminate thyroid nodules. J Endocrinol Invest.

[34] Nikiforov YE, Ohori NP, Hodak SP, Carty SE, LeBeau SO, Ferris RL, Yip L, Seethala RR, Tublin ME, Stang MT, Coyne C, Johnson JT, Stewart AF, Nikiforova MN (2011). Impact of mutational testing on the diagnosis and management of patients with cytologically indeterminate thyroid nodules: a prospective analysis of 1056 FNA samples. J Clin Endocrinol Metab.

[35] Beaudenon-Huibregtse S, Alexander EK, Guttler RB, Hershman JM, BabuV, Blevins TC, Moore P, Andruss B, Labourier E (2014) Centralized molecular testing for oncogenic gene mutations complements the local cytopathologic diagnosis of thyroid nodules. Thyroid 24:1479–1487 10.1089/thy.2013.0640 24811481

[36] Eszlinger M, Böhme K, Ullmann M, Görke F, Siebolts U, Neumann A, Franzius C, Adam S, Molwitz T, Landvogt C, Amro B, Hach A, Feldmann B, Graf D, Wefer A, Niemann R, Bullmann C, Klaushenke G, Santen R, Tönshoff G, Ivancevic V, Kögler A, Bell E, Lorenz B, Kluge G, Hartenstein C, Ruschenburg I, Paschke R (2017). Evaluation of a 2-year routine application of molecular testing of thyroid fine-needle aspirations using a seven-gene panel in a primary referral setting in Germany. Thyroid.

[37] Fnais N, Soobiah C, Al-Qahtani K, Hamid JS, Perrier L, Straus SE, Tricco AC (2015). Diagnostic value of fine needle aspiration BRAF(V600E) mutation analysis in papillary thyroid cancer: a systematic review and meta-analysis. Hum Pathol.

[38] Rossi M, Buratto M, Tagliati F, Rossi R, Lupo S, Trasforini G, Lanza G, Franceschetti P, Bruni S, Degli Uberti E, Zatelli MC (2015). Relevance of BRAF(V600E) mutation testing versus RAS point mutations and RET/PTC rearrangements evaluation in the diagnosis of thyroid cancer. Thyroid.

[39] Rossi M, Lupo S, Rossi R, Franceschetti P, Trasforini G, Bruni S, Tagliati F, Buratto M, Lanza G, Damiani L, Degli Uberti E, Zatelli MC (2017). Proposal for a novel management of indeterminate thyroid nodules on the basis of cytopathological subclasses. Endocrine.

