# Supplementary material for: Combination of ultrasound and molecular testing in malignancy risk estimate of Bethesda category IV thyroid nodules: results from a single-institution prospective study
Source: J Endocrinol Invest. 2021 Apr 16;44(12):2635–43. doi: 10.1007/s40618-021-01571-y (PMC8572191; doi:10.1007/s40618-021-01571-y)
Supplement: Supplementary file 1 — Supplementary file1 (DOCX 14 KB) [file 40618_2021_1571_MOESM1_ESM.docx]

Supplementary Table 1

Custom Dual Labelled Probe and oligonucleotide sequences for genotyping RET/PTC1 and RET/PTC3 gene rearrangements (Metabion International AG)

| RET/PTC1 Probe | 6-Fam-AAC CGC GAC CTG CGC AAA GC-BHQ-1 |
| --- | --- |
| RET/PTC1-FOR primer | 5’-GGA GAC CTA CAA ACT GAA GTG CAA-3’ |
| RET/PTC1-REV primer | 5’-CCC TTC TCC TAG AGT TTT TCC AAG A-3’ |
| RET/PTC3 Probe | 6-Fam-ACC CAG CAC CGA CCC CCA GG-BHQ-1 |
| RET/PTC3-FOR primer | 5’-CCA GTG GTT ATC AAG CTC CTT ACA-3’ |
| RET/PTC3-REV primer | 5’-GGG AAT TCC CAC TTT GGA TCC TC-3’ |
